# Outcomes Associated With Continuous Renal Replacement Therapy in Acute Respiratory Distress Syndrome With Acute Kidney Injury: A National Inpatient Sample Analysis

**DOI:** 10.7759/cureus.112363

**Published:** 2026-07-09

**Authors:** Abdelhadi Farouji, Muhammed Umer, Mu'ath Abdeen, Masara Touza, Ratib Mahfouz

**Affiliations:** 1 Division of Nephrology, Department of Internal Medicine, Henry Ford Hospital, Detroit, USA; 2 Department of Internal Medicine, Saint Michael’s Medical Center, New York Medical College, Newark, USA; 3 Department of Internal Medicine, Trinity Health Livonia, Livonia, USA; 4 Division of Nephrology, Department of Internal Medicine, Cleveland Clinic, Cleveland, USA

**Keywords:** acute kidney injury (aki), acute respiratory distress syndrome (ards), continuous renal replacement therapy (crrt), in-hospital mortality, national inpatient sample (nis)

## Abstract

Background

Acute respiratory distress syndrome (ARDS) is frequently complicated by acute kidney injury (AKI) and is associated with adverse outcomes. Continuous renal replacement therapy (CRRT) is commonly used in critically ill patients with hemodynamic instability; however, nationally representative data evaluating outcomes associated with CRRT use in ARDS complicated by AKI are limited.

Materials and methods

We conducted a retrospective cohort study using the Healthcare Cost and Utilization Project National Inpatient Sample (HCUP-NIS) from 2016 to 2021. Adult hospitalizations with ARDS and AKI were identified using ICD-10-CM codes. Patients with end-stage renal disease were excluded. Multivariable logistic regression analyses were performed to evaluate the association between CRRT use and in-hospital outcomes, adjusting for demographics, comorbidities, hospital characteristics, and primary payer. Discharge weights were applied to generate nationally representative estimates.

Results

Among an estimated 485,035 hospitalizations with ARDS and AKI, 26,995 (5.6%) patients received CRRT. Unadjusted in-hospital mortality was significantly higher among patients receiving CRRT compared with those not receiving CRRT (77.7% vs. 43.1%, p < 0.001). After multivariable adjustment, CRRT use remained associated with higher odds of in-hospital mortality (adjusted odds ratio [aOR] 3.30; 95% CI 3.06-3.55) and higher odds of mechanical ventilation, cardiac arrest, sepsis, vasopressor use, disseminated intravascular coagulation, extracorporeal membrane oxygenation use, and arrhythmia (all p < 0.001).

Conclusions

Because patients requiring CRRT likely represent those with greater illness severity and because physiologic severity measures (e.g., Sequential Organ Failure Assessment [SOFA] and Acute Physiology and Chronic Health Evaluation [APACHE] scores) are unavailable in the HCUP-NIS, these associations should be interpreted cautiously and likely reflect confounding by indication and residual confounding rather than a direct causal effect of CRRT. CRRT use was associated with substantially higher in-hospital mortality, complication burden, and healthcare resource utilization, identifying a subgroup of patients with severe multisystem organ failure and poor short-term prognosis.

## Introduction

Acute respiratory distress syndrome (ARDS) remains a major cause of morbidity and mortality among critically ill patients worldwide [1]. Despite advances in lung-protective ventilation strategies and supportive care, ARDS continues to be associated with prolonged intensive care unit (ICU) stays, substantial healthcare resource utilization, and high in-hospital mortality in contemporary cohorts [2]. The systemic inflammatory response and hemodynamic instability characteristic of severe disease frequently lead to extrapulmonary organ dysfunction, further complicating management and adversely affecting outcomes [1,2].

Acute kidney injury (AKI) is among the most common and clinically significant complications in this population and has been consistently associated with increased mortality [3]. Prior studies have demonstrated that AKI in ARDS is independently associated with worse clinical outcomes, particularly among patients who progress to severe kidney failure requiring renal replacement therapy [4,5]. Continuous renal replacement therapy (CRRT) is commonly utilized in this setting due to its suitability for patients with hemodynamic instability, fluid overload, and multiorgan failure. Patients requiring CRRT represent a subgroup with more severe renal dysfunction, greater physiologic instability, and advanced multiorgan failure. Accordingly, CRRT use is more appropriately interpreted as a marker of illness severity rather than an independent determinant of outcomes.

Despite prior studies evaluating renal replacement therapy in ARDS or severe AKI, most have been limited by single-center designs, relatively small sample sizes, or disease-specific populations. Contemporary, nationally representative data evaluating in-hospital mortality, complication burden, discharge disposition, and healthcare resource utilization associated with CRRT use in a broad, nationally representative population of patients hospitalized with ARDS and AKI in the United States remain limited. Using the Healthcare Cost and Utilization Project National Inpatient Sample (HCUP-NIS), we conducted a large, nationally representative retrospective cohort study to compare in-hospital outcomes between patients with ARDS complicated by AKI who received CRRT and those who did not. Our objective was to evaluate the association between CRRT use and in-hospital outcomes and to characterize the clinical phenotype of patients requiring CRRT. Given the observational nature of the HCUP-NIS and the absence of temporal and physiologic severity data, causal inference is not possible, and CRRT should be interpreted primarily as a marker of illness severity rather than a determinant of outcomes.

## Materials and methods

Data source

This retrospective cohort study used data from the HCUP-NIS for the years 2016 through 2021. The NIS is sponsored by the Agency for Healthcare Research and Quality (AHRQ) and represents the largest publicly available all-payer inpatient healthcare database in the United States.

The NIS consists of an approximately 20% stratified sample of inpatient discharges from participating U.S. states and is weighted to generate nationally representative estimates, capturing more than 97% of U.S. inpatient hospitalizations after applying discharge weights. The sampling strategy is based on hospital characteristics including geographic region, location, teaching status, bed size, and ownership.

Each hospitalization record includes one principal diagnosis, multiple secondary diagnoses, and procedure codes defined using the International Classification of Diseases, Tenth Revision, Clinical Modification and Procedure Coding System (ICD-10-CM/PCS). Because the NIS contains de-identified publicly available data, this study was exempt from institutional review board approval and informed consent requirements.

The NIS database does not provide information regarding the timing or sequence of clinical events, including the onset of ARDS, development of AKI, initiation of CRRT, or the occurrence of sepsis, vasopressor use, and other in-hospital complications. Consequently, the temporal relationship between these events cannot be determined, precluding assessment of whether they preceded or followed CRRT initiation and limiting causal interpretation of the observed associations.

Study variables

The study population included adult patients aged 18 years and older. Using ICD-10-CM and ICD-10-PCS codes, we identified hospitalizations with ARDS, AKI, and receipt of CRRT. The complete ICD-10-CM and ICD-10-PCS codes used to define the study population, comorbidities, procedures, and clinical outcomes are provided in Table 1.

**Table 1:** ICD-10-CM and ICD-10-PCS codes used to define the study population, comorbidities, procedures, and clinical outcomes. AKI, acute kidney injury; ARDS, acute respiratory distress syndrome; ESRD, end-stage renal disease; CRRT, continuous renal replacement therapy; DIC, disseminated intravascular coagulation; ECMO, extracorporeal membrane oxygenation

Variables	ICD-10 code(s)
ARDS	J80
AKI	N17.x
ESRD	N18.6
CRRT	5A1D60Z, 5A1D80Z, 5A1D90Z
Diabetes mellitus	E10.x, E11.x, E13.x
Hypertension	I10–I15
Chronic kidney disease (stage ≥3)	N18.3–N18.5
Congestive heart failure	I50.x
Coronary artery disease	I25.x, Z95.1, Z95.5
Chronic obstructive pulmonary disease	J44.x
Pulmonary hypertension	I27.x
Liver cirrhosis	K70.30, K70.31, K74.60, K74.69
Mechanical ventilation	5A1945Z, 5A1955Z
Vasopressor use	3E033XZ
ECMO use	5A15223, 5A1522F, 5A1522G, 5A1522H
Sepsis	A40.x, A41.x
Cardiac arrest	I46.x
DIC	D65
Arrhythmia	I47.x, I48.x, I49.x
Tracheostomy	0B110F4, 0B113F4

Baseline patient characteristics included age (years), sex, race/ethnicity (White, Black, Hispanic, and Other) as reported in the NIS, primary payer status, and hospital characteristics (bed size). Comorbid conditions were identified using individual ICD-10-CM code lists and included diabetes mellitus, hypertension, chronic kidney disease stage ≥3, congestive heart failure, coronary artery disease, chronic obstructive pulmonary disease, pulmonary hypertension, and liver cirrhosis. Patients with end-stage renal disease (ESRD) were excluded using ICD-10-CM code N18.6 to minimize misclassification of chronic dialysis as CRRT.

Statistical analysis

Statistical analyses were performed using Stata version 17.0 (StataCorp, College Station, TX, USA). Baseline demographic and clinical characteristics were summarized using descriptive statistics and compared between ARDS patients who received CRRT and those who did not.

Continuous variables were summarized as mean ± standard deviation (SD) and compared using Student’s t-test. Categorical variables were expressed as frequencies and percentages and compared using the Pearson χ² (chi-square) test.

Multivariable logistic regression models were prespecified based on clinically relevant covariates and prior literature to evaluate the association between CRRT use and in-hospital outcomes. Covariates included age, sex, race/ethnicity, primary payer status, hospital bed size, diabetes mellitus, hypertension, chronic kidney disease stage ≥3, congestive heart failure, coronary artery disease, chronic obstructive pulmonary disease, pulmonary hypertension, and liver cirrhosis. Because the HCUP-NIS does not capture physiologic severity measures, residual confounding related to illness severity may persist despite multivariable adjustment, and causal inference cannot be established.

Adjusted odds ratios (aORs) with 95% confidence intervals (CIs) were reported. A two-tailed p-value of <0.05 was considered statistically significant. All analyses incorporated NIS discharge weights to generate nationally representative estimates.

The NIS dataset contains minimal missing data for key variables; analyses were conducted using complete-case methodology. All statistical tests were two-sided.

## Results

Demographic and clinical characteristics

A total of 485,035 adult hospitalizations with ARDS and AKI, after exclusion of patients with ESRD, were identified from the NIS database. Among these, 26,995 (5.57%) patients received CRRT, while 458,040 (94.43%) patients did not receive CRRT (Table 2).

**Table 2:** Study cohort selection of adult hospitalizations with ARDS and AKI from the National Inpatient Sample (2016–2021). AKI, acute kidney injury; ARDS, acute respiratory distress syndrome; ESRD, end-stage renal disease

Study cohort selection	Number of hospitalizations
Adult hospitalizations with ARDS and AKI identified from the NIS database (2016–2021)	503,281
Excluded: ESRD	18,246
Final cohort of hospitalizations with ARDS complicated by AKI	485,035

Patients who received CRRT were younger than those who did not (mean age 58.41 ± 14.07 vs. 60.68 ± 16.10 years; p < 0.001), and male sex predominated in the CRRT group (65.46% [n=17,672] vs. 56.02% [n=256,663], p < 0.001).

With respect to race and ethnicity, White patients constituted the largest proportion in both cohorts but were less frequently represented in the CRRT group (50.31% [n=13,580] vs. 57.88% [n=265,086], p < 0.001). Black patients were more frequently represented among those receiving CRRT (20.84% [n=5,625] vs. 13.80% [n=63,209], p < 0.001), while the proportion of Hispanic patients was similar between groups.

Regarding comorbidities, patients in the CRRT group had a higher prevalence of diabetes mellitus, chronic kidney disease stage ≥3, congestive heart failure, and liver cirrhosis. Coronary artery disease and chronic obstructive pulmonary disease were more prevalent in the non-CRRT group, whereas no statistically significant differences were observed in the prevalence of hypertension or pulmonary hypertension. These baseline differences suggest that patients requiring CRRT represented a clinically more complex population with greater underlying illness burden rather than indicating an association between CRRT and the presence of these comorbidities.

In terms of hospital characteristics, CRRT use was more common in large hospitals (70.03% [n=18,904] vs. 52.22% [n=239,290], p < 0.001). Regarding primary payer status, patients receiving CRRT were less likely to be insured by Medicare and more likely to have Medicaid or private insurance compared with those not receiving CRRT (Table 3).

**Table 3:** Demographic and clinical characteristics of patients with ARDS and AKI receiving CRRT versus those not receiving CRRT. Values are presented as mean ± standard deviation (SD) or weighted percentages with corresponding counts (n). P-values were calculated using Student’s t-test for continuous variables and Pearson χ² tests for categorical variables, as appropriate. All analyses incorporated NIS discharge weights to generate nationally representative estimates. AKI, acute kidney injury; ARDS, acute respiratory distress syndrome; CRRT, continuous renal replacement therapy; NIS, National Inpatient Sample

Variable	ARDS + AKI with CRRT (n=26,995)	ARDS + AKI without CRRT (n=458,040)	p-Value
Age, mean ± SD (years)	58.41 ± 14.07	60.68 ± 16.10	<0.001
Sex, %			
Female	34.54 (n=9,323)	43.98 (n=201,377)	<0.001
Male	65.46 (n=17,672)	56.02 (n=256,663)	
Race, %			
White	50.31 (n=13,580)	57.88 (n=265,086)	<0.001
Black	20.84 (n=5,625)	13.80 (n=63,209)	
Hispanic	16.71 (n=4,512)	16.69 (n=76,441)	
Other	12.15 (n=3,278)	11.63 (n=53,304)	
Comorbidities, %			
Diabetes mellitus	40.41 (n=10,905)	37.35 (n=171,163)	<0.001
Hypertension	57.88 (n=15,625)	59.24 (n=271,372)	0.081
Chronic kidney disease (stage ≥3)	27.91 (n=7,535)	18.15 (n=83,167)	<0.001
Congestive heart failure	25.49 (n=6,881)	23.27 (n=106,567)	<0.001
Coronary artery disease	14.37 (n=3,877)	16.72 (n=76,585)	<0.001
Chronic obstructive pulmonary disease	13.82 (n=3,732)	18.84 (n=86,291)	<0.001
Pulmonary hypertension	6.26 (n=1,689)	5.78 (n=26,475)	0.169
Liver cirrhosis	6.96 (n=1,878)	3.23 (n=14,797)	<0.001
Hospital bed size, %			
Small	11.76 (n=3,174)	20.20 (n=92,523)	<0.001
Medium	18.21 (n=4,917)	27.57 (n=126,227)	
Large	70.03 (n=18,904)	52.22 (n=239,290)	
Primary payer, %			
Medicare	39.90 (n=10,770)	45.64 (n=209,085)	<0.001
Medicaid	19.21 (n=5,185)	17.52 (n=80,244)	
Private insurance	32.44 (n=8,759)	28.42 (n=130,201)	
Self-pay	4.19 (n=1,131)	4.20 (n=19,238)	
Other/No charge	4.25 (n=1,150)	4.23 (n=19,272)	

In-hospital mortality

Unadjusted in-hospital mortality was significantly higher among patients with ARDS and AKI who received CRRT compared with those who did not (77.71% [n=20,986] vs. 43.07% [n=197,420], p < 0.001).

After multivariable adjustment for demographics, comorbidities, and hospital characteristics, CRRT use remained associated with higher odds of in-hospital mortality (aOR, 3.30; 95% CI, 3.06-3.55; p < 0.001) (Table 4). Because the HCUP-NIS lacks physiologic severity measures, including Sequential Organ Failure Assessment (SOFA) or Acute Physiology and Chronic Health Evaluation (APACHE) scores, PaO₂/FiO₂ ratio, vasopressor dose, lactate concentration, and fluid balance, residual confounding related to illness severity may persist despite adjustment, and the observed association should not be interpreted as causal.

**Table 4:** In-hospital outcomes and discharge disposition among patients with ARDS and AKI receiving CRRT versus those not receiving CRRT. Discharge disposition percentages were calculated among patients who survived hospitalization. P-values were calculated using Student’s t-test for continuous variables and Pearson χ² tests for categorical variables, as appropriate. Analyses incorporated NIS discharge weights. AKI, acute kidney injury; ARDS, acute respiratory distress syndrome; CRRT, continuous renal replacement therapy; NIS, National Inpatient Sample; SNF/ICF, skilled nursing facility/intermediate care facility

Outcome	ARDS + AKI with CRRT (n=26,995)	ARDS + AKI without CRRT (n=458,040)	P-Value
In-hospital mortality, %	77.71 (n=20,986)	43.07 (n=197,420)	<0.001
Length of stay, days, mean ± SD	22.7 ± 19.6	17.3 ± 16.7	<0.001
Total hospital charges, $, mean ± SD	557,425 ± 657,753	295,427 ± 421,567	<0.001
Discharge disposition among survivors, %			
Routine discharge to home	12.16 (n=731)	33.26 (n=86,695)	<0.001
Home healthcare	9.47 (n=569)	16.86 (n=43,945)	
Transfer to short-term hospital	9.92 (n=596)	10.72 (n=27,942)	
Transfer to SNF/ICF/other facility	67.96 (n=4,083)	37.92 (n=98,844)	
Left against medical advice	0.49 (n=29)	1.24 (n=3,232)	

Other in-hospital outcomes and discharge disposition

Compared with patients who did not receive CRRT, those receiving CRRT had a longer mean length of stay (22.7 vs. 17.3 days, p < 0.001) and higher mean total hospital charges ($557,425 vs. $295,427, p < 0.001).

Regarding discharge disposition among survivors, patients in the CRRT group were less likely to be routinely discharged home (12.16% vs. 33.26%, p < 0.001) or to receive home healthcare (9.47% vs. 16.86%, p < 0.001). Transfers to short-term hospitals were similar between groups (9.92% vs. 10.72%), whereas transfers to skilled nursing facilities, intermediate care facilities, or other institutions were more common among patients receiving CRRT (67.96% vs. 37.92%, p < 0.001). Leaving against medical advice was uncommon in both groups but occurred less frequently in the CRRT group (0.49% vs. 1.24%, p < 0.001).

Discharge categories were mutually exclusive, defined according to NIS disposition variables, and calculated among patients who survived hospitalization (Table 4).

Clinical complications during hospitalization

In unadjusted analyses, patients receiving CRRT had higher rates of in-hospital complications compared with those who did not (Table 5). The CRRT group had higher rates of sepsis, mechanical ventilation, vasopressor use, extracorporeal membrane oxygenation (ECMO), cardiac arrest, arrhythmias, disseminated intravascular coagulation (DIC), and tracheostomy (all p < 0.001). Because the HCUP-NIS does not capture the timing of clinical events, it cannot be determined whether these complications preceded, coincided with, or followed CRRT initiation; therefore, these findings should not be interpreted as evidence of a temporal or causal relationship.

**Table 5:** Comparison of clinical complications during hospitalization among patients with ARDS and AKI receiving CRRT versus those not receiving CRRT. Values are presented as weighted percentages with corresponding counts (n). P-values were calculated using Pearson χ² tests for categorical variables. Analyses incorporated NIS discharge weights. AKI, acute kidney injury; ARDS, acute respiratory distress syndrome; CRRT, continuous renal replacement therapy; DIC, disseminated intravascular coagulation; ECMO, extracorporeal membrane oxygenation; NIS, National Inpatient Sample

Clinical complications, % (n)	ARDS + AKI with CRRT	ARDS + AKI without CRRT	p-Value
Mechanical ventilation	96.67 (n=26,105)	63.10 (n=289,028)	<0.001
Sepsis	87.91 (n=23,741)	58.12 (n=266,187)	<0.001
Vasopressor use	37.91 (n=10,240)	13.24 (n=60,645)	<0.001
ECMO	9.76 (n=2,635)	2.52 (n=11,545)	<0.001
Cardiac arrest	17.71 (n=4,782)	10.00 (n=45,804)	<0.001
Arrhythmia	42.93 (n=11,591)	30.83 (n=141,256)	<0.001
DIC	9.06 (n=2,446)	2.34 (n=10,719)	<0.001
Tracheostomy	16.54 (n=4,463)	10.01 (n=45,850)	<0.001

After multivariable adjustment for patient demographics, comorbidities, and hospital characteristics, CRRT use remained associated with higher odds of multiple in-hospital complications. Specifically, CRRT use was associated with higher odds of mechanical ventilation (aOR, 6.63; 95% CI, 5.69-7.73), cardiac arrest (aOR, 5.10; 95% CI, 4.81-5.39), sepsis (aOR, 2.67; 95% CI, 2.45-2.91), vasopressor use (aOR, 2.23; 95% CI, 2.10-2.37), DIC (aOR, 2.98; 95% CI, 2.69-3.31), tracheostomy (aOR, 1.66; 95% CI, 1.53-1.80), ECMO use (aOR, 1.59; 95% CI, 1.45-1.74), and arrhythmia (aOR, 1.25; 95% CI, 1.21-1.30), all p < 0.001 (Table 6). Because the HCUP-NIS does not capture the temporal sequence of clinical events or physiologic severity, these associations should not be interpreted as evidence that CRRT independently contributes to these complications but rather likely reflect the greater severity of illness and need for multiple organ support among patients requiring CRRT.

**Table 6:** Multivariable logistic regression analysis of the association between CRRT use and in-hospital outcomes among patients with ARDS and AKI. aORs represent the association between CRRT use and each outcome after adjustment for age, sex, race/ethnicity, primary payer, hospital bed size, diabetes mellitus, hypertension, chronic kidney disease stage ≥3, congestive heart failure, coronary artery disease, chronic obstructive pulmonary disease, pulmonary hypertension, and liver cirrhosis. The reference group consisted of patients with ARDS and AKI who did not receive CRRT. AKI, acute kidney injury; aOR, adjusted odds ratio; ARDS, acute respiratory distress syndrome; CRRT, continuous renal replacement therapy; DIC, disseminated intravascular coagulation; ECMO, extracorporeal membrane oxygenation

Outcome	aOR	95% Confidence Interval	p-Value
In-hospital mortality	3.30	3.06–3.55	<0.001
Mechanical ventilation	6.63	5.69–7.73	<0.001
Vasopressor use	2.23	2.10–2.37	<0.001
ECMO use	1.59	1.45–1.74	<0.001
Sepsis	2.67	2.45–2.91	<0.001
Cardiac arrest	5.10	4.81–5.39	<0.001
DIC	2.98	2.69–3.31	<0.001
Arrhythmia	1.25	1.21–1.30	<0.001
Tracheostomy	1.66	1.53–1.80	<0.001

## Discussion

In this large, contemporary, nationally representative cohort of patients hospitalized with ARDS and AKI in the United States, approximately 5.6% required CRRT. Patients requiring CRRT represented a subgroup with more severe critical illness and experienced substantially higher in-hospital mortality, greater clinical complication burden, longer hospital stays, and higher healthcare resource utilization than those who did not receive CRRT. These associations persisted after adjustment for patient demographics, comorbid conditions, hospital characteristics, and payer status. Importantly, these findings should not be interpreted as evidence that CRRT itself contributes to adverse outcomes. Rather, CRRT use likely reflects a subgroup of patients with more severe underlying illness, characterized by advanced multiorgan failure, hemodynamic instability, and greater physiologic derangement [4]. Accordingly, CRRT should be interpreted primarily as a marker of critical illness severity rather than an independent determinant of prognosis [4].

The observed associations between CRRT use and adverse in-hospital outcomes most likely reflect the severity of underlying critical illness rather than direct adverse effects of CRRT itself [5]. Patients with ARDS complicated by AKI who require CRRT represent a population with severe renal dysfunction occurring in the setting of profound respiratory failure, systemic inflammation, and hemodynamic instability [4,5]. Experimental and clinical data support a bidirectional kidney-lung interaction in critical illness, whereby inflammatory mediator release, endothelial dysfunction, and microvascular injury contribute to concurrent pulmonary and renal failure [5]. AKI may further exacerbate lung injury through impaired cytokine clearance, dysregulated fluid balance, and increased capillary permeability, while severe hypoxemia and positive-pressure ventilation can compromise renal perfusion and oxygen delivery [3,5]. Cumulative fluid overload has been independently associated with worse oxygenation, prolonged mechanical ventilation, and increased mortality in ARDS and septic shock populations [6,7]. Hemodynamic instability and vasodilatory shock - most commonly driven by sepsis - frequently necessitate vasopressor support and favor the use of CRRT in patients with multiorgan failure [8,9]. Consistent with this pathophysiologic framework, sepsis was highly prevalent among patients receiving CRRT in our cohort, supporting the interpretation that CRRT use identifies an extreme critical illness phenotype characterized by simultaneous respiratory, renal, and circulatory failure. Importantly, given the limitations of administrative data, the temporal relationship between these complications and CRRT initiation cannot be determined.

Prior investigations have consistently demonstrated that the development of AKI, particularly when severe enough to require renal replacement therapy, is associated with markedly worse outcomes among critically ill patients with respiratory failure and ARDS; however, important gaps remain in the existing literature [4]. Much of the available evidence is derived from single-center cohorts or heterogeneous ICU populations with mixed etiologies of respiratory failure, thereby limiting external validity and the ability to define population-level risk [4,8]. ARDS-focused studies evaluating renal replacement therapy have largely been confined to specific disease contexts [4]. In an influenza pneumonia-related ARDS cohort, Chang et al. demonstrated substantially increased mortality among patients requiring renal replacement therapy; however, the study was limited by its relatively small sample size and disease-specific scope, thereby restricting generalizability to the broader ARDS population [4]. In parallel, mechanistic and translational studies have established a bidirectional relationship between lung and kidney injury mediated by systemic inflammation, endothelial dysfunction, and disordered fluid balance, providing biological plausibility for the poor outcomes observed in patients with concurrent ARDS and severe AKI, although these studies do not quantify outcomes at a population level once CRRT is initiated [3,5]. Against this background, the present study extends prior work by leveraging a large, contemporary, nationally representative U.S. cohort of patients hospitalized with ARDS to provide national-level estimates of outcomes associated with CRRT use [1-5]. By examining not only in-hospital mortality but also a broad spectrum of clinical complications, discharge disposition, length of stay, and hospital charges, our analysis provides a comprehensive and generalizable assessment of the clinical and healthcare-system burden associated with CRRT use across diverse hospitals and practice settings. Importantly, the primary contribution of this study is to provide nationally representative estimates of these associations rather than to establish new mechanistic insights or causal effects of CRRT, which cannot be inferred from this observational analysis [1-5].

In adjusted analyses, CRRT use among patients with ARDS complicated by AKI remained associated with substantially higher odds of multiple critical care interventions and adverse in-hospital outcomes, including mechanical ventilation, vasopressor use, ECMO, and cardiac arrest. Because the HCUP-NIS does not capture the temporal sequence of clinical events or physiologic severity measures, these associations likely reflect the preferential use of CRRT in patients with advanced multisystem organ failure who already require multiple organ support, rather than indicating that CRRT itself increases the risk of these complications. Rather, these findings likely reflect a distinct critical illness phenotype characterized by advanced, multisystem organ failure [9]. Patients with ARDS complicated by AKI who require CRRT frequently exhibit concurrent respiratory, circulatory, and hematologic dysfunction, often driven by severe sepsis or septic shock, necessitating escalating levels of organ support [10,11]. Within this context, CRRT represents one component of a broader life-sustaining treatment strategy rather than an isolated marker of renal injury, consistent with established frameworks describing organ dysfunction and failure in critically ill patients [9-11]. The consistent association between CRRT use and markers of extreme physiologic derangement across multiple organ systems underscores its role as an indicator of profound illness severity and helps explain the markedly elevated mortality observed in this population [4,8].

CRRT use among patients with ARDS complicated by AKI was associated with substantially increased healthcare resource utilization and unfavorable discharge outcomes [4,8]. Patients receiving CRRT experienced significantly longer hospital lengths of stay and nearly twofold higher total hospital charges compared with those who did not receive CRRT, reflecting the intensity and duration of critical care required in this population [8-10]. Favorable discharge outcomes were uncommon, with routine discharge to home occurring in approximately 12% of survivors, and the majority requiring transfer to post-acute care facilities or experiencing in-hospital mortality [4]. These findings underscore the considerable systems-level burden associated with CRRT use in ARDS and highlight its implications for ICU resource planning, staffing, and bed allocation [8-10]. From a clinical perspective, recognition that CRRT use identifies an exceptionally high-risk subgroup may facilitate timely prognostication, multidisciplinary care coordination, and goals-of-care discussions with patients and families, particularly in the setting of prolonged ICU courses and limited likelihood of functional recovery [8-11].

This study has several important limitations that merit consideration. First, as a retrospective observational analysis of administrative data, causal inferences cannot be made, and the observed associations between CRRT use and adverse outcomes are subject to residual confounding, particularly confounding by indication. Patients receiving CRRT likely represent those with the greatest physiologic severity, and although we adjusted for a broad range of demographic, comorbidity, and hospital-level factors, substantial residual confounding due to unmeasured illness severity, including the absence of physiologic severity measures such as SOFA or APACHE scores, PaO₂/FiO₂ ratio, vasopressor dose, lactate concentration, and cumulative fluid balance, likely remains. Accordingly, the adjusted associations should not be interpreted as evidence of independent causal effects of CRRT. Second, the HCUP-NIS lacks granular clinical and physiologic data, including PaO₂/FiO₂ ratio, severity-of-illness scores (e.g., SOFA or APACHE), vasopressor dose, ventilator parameters, and cumulative fluid balance, limiting our ability to fully characterize illness severity. Third, the HCUP-NIS does not capture details regarding the timing, indication, modality, or dose of CRRT. Consequently, treatment sequencing and the temporal relationships among ARDS, AKI, sepsis, vasopressor initiation, and CRRT initiation could not be determined, substantially limiting causal interpretation of the observed associations. Fourth, reliance on ICD-10 diagnosis and procedure codes introduces the potential for misclassification and variability in coding practices across institutions. Fifth, CRRT availability is more common in large and tertiary care centers, which may introduce residual confounding related to hospital type and referral patterns despite adjustment for hospital characteristics. Sixth, the study period (2016-2021) includes the COVID-19 pandemic, during which ARDS epidemiology and management evolved substantially, potentially influencing the observed outcomes. Finally, the HCUP-NIS captures in-hospital events only; therefore, long-term outcomes, including post-discharge mortality and functional recovery, could not be assessed.

Despite these limitations, the large sample size, contemporary timeframe, and nationally representative design provide robust population-level insight, enhance generalizability, and allow comprehensive evaluation of clinical outcomes, complications, and healthcare resource utilization associated with CRRT use among patients with ARDS in the United States.

## Conclusions

In this large, contemporary, nationally representative analysis of patients hospitalized with ARDS and AKI in the United States, CRRT use was associated with very high in-hospital mortality, a substantial burden of clinical complications, and markedly increased healthcare resource utilization. These associations likely reflect the greater underlying illness severity of patients requiring CRRT rather than effects attributable to CRRT itself. CRRT use identified a subgroup of patients with ARDS characterized by advanced multisystem organ failure and a very poor short-term prognosis. These findings emphasize the importance of recognizing CRRT use as a marker of severe illness in ARDS. Future prospective studies incorporating granular physiologic and severity-of-illness data are warranted to refine risk stratification and better define the optimal timing and indications for CRRT in this high-risk population.
